# Changes in 10-12 year old's fruit and vegetable intake in Norway from 2001 to 2008 in relation to gender and socioeconomic status - a comparison of two cross-sectional groups

**DOI:** 10.1186/1479-5868-8-108

**Published:** 2011-10-03

**Authors:** Marit Hilsen, Maartje M van Stralen, Knut-Inge Klepp, Elling Bere

**Affiliations:** 1 Faculty of Health and Sport, University of Agder, Norway; 2 Department of Nutrition, Faculty of Medicine, University of Oslo, Norway; 3 Department of Public and Occupational Health, the EMGO Institute for Health and Care Research, VU University Medical Center, the Netherlands

**Keywords:** Fruit and vegetable intake, time trends, gender, socioeconomic status, children

## Abstract

**Background:**

Norwegian children and adolescents eat less than half of the recommended 5 portions of fruit and vegetables (FV) per day. Gender and socioeconomic disparities in FV consumption shows that boys and children of lower socioeconomic status (SES) eat less FV than girls and high SES children. We also know that accessibility and preferences has been identified as two important determinants of FV intake. The objectives of this study were to compare FV intake among Norwegian 6^th ^and 7^th ^graders in 2001 and 2008, to explore potential mediated effects of accessibility and preferences on changes in FV over time, to explore whether these changes in FV intake was moderated by gender and/or SES and whether a moderated effect in FV intake was mediated by accessibility and preferences of FV.

**Methods:**

The baseline survey of the Fruits and Vegetables Make the Marks project was conducted in 2001 at 38 randomly chosen schools in two Norwegian counties. A second survey was conducted at the same schools in 2008. A total of 27 schools participated in both surveys (2001 n = 1488, 2008 n = 1339). FV intake was measured by four food frequency questions (times/week) in a questionnaire which the pupils completed at school. SES was based on parents' reports of their own educational level in a separate questionnaire. The main analyses were multilevel linear regression analyses.

**Results:**

A significant year*parental educational level interaction was observed (p = 0.01). FV intake decreased among pupils of parents with lower educational level (13.9 vs. 12.6 times/week in 2001 and 2008, respectively), but increased among pupils of parents with higher education (14.8 vs. 15.0 times/week, respectively). This increasing SES disparity in FV intake was partly mediated by an increasing SES disparity in accessibility and preferences over time, wherein children with higher educated parents had a steeper increase in accessibility and preferences over time than children with lower educated parents. The year*sex interaction was not significant (p = 0.54).

**Conclusions:**

This study shows an increase in SES disparities in 6^th ^and 7^th ^graders FV intake from 2001 to 2008, partly mediated by an increasing SES disparity in accessibility and preferences of FV.

## Background

Research shows that a diet high in fruits and vegetables (FV) reduces the risk of developing several chronic diseases [1] and that food habits and preferences established during childhood and adolescents track well into adulthood [2,3]. Childhood is therefore a crucial time point to initiate lifelong healthy eating habits and thereby achieve a maximum preventive effect against diet related chronic diseases. However, data shows that less than 50% of Norwegian 8^th ^graders consume the recommended intake of FV per day. Only 11% of the 8^th ^graders consumed more than 500 grams FV per day and the mean FV intake among 8^th ^graders was 255 grams per day [4,5].

Norway is a welfare state with a high gross domestic product (GDP) per capita. However, social disparities, including health behaviour and outcome, is evident in Norway [6]. E.g. food choices have been reported to follow a socioeconomic gradient indicating that groups of higher socioeconomic status (SES) consume healthy food items more frequently than individuals of lower SES [7]. These gradients have also been observed among children and adolescents [4,8,9]. In addition to social disparities in food choices, gender differences have also been observed indicating that girls report to eat more FV than boys [9].

Beyond socioeconomic and gender differences the aetiology of food behaviour may be further understood by studying determinants of FV intake. Modifiable determinants such as accessibility and preferences have, in addition to being correlated (r = 0.43 and r = 0.45, respectively) to FV intake [10], also been reported to be among the strongest predictors to explain future FV intake among schoolchildren [11]. Previous longitudinal analysis within the Fruits and Vegetables Make the Marks (FVMM) cohort project showed that perceived accessibility alone explained 90% (age 12.5) and 50% (age 15.5) of parental educational disparities [8], and preferences alone explained 81% of the gender disparities [12] observed in FV intake among adolescents.

The Norwegian government has aimed at reducing the social disparities in health behaviour and outcome by several initiatives [13] including nutritional guidelines [14,15]. Due to these governmental efforts it is of great interest to study the most recent development in health related trends. Data on the development of gender and socioeconomic disparities in eating habits over the last years is scarce and the need for such results are therefore called for in order to tailor effective interventions in the future.

The objectives of this study were to compare FV intake among Norwegian 6^th ^and 7^th ^graders in 2001 and 2008, to explore potential mediated effects of accessibility and preferences on changes in FV over time, to explore whether these changes in FV intake was moderated by gender and/or SES and whether this moderating effect on changes in FV intake over time was mediated by accessibility and preferences of FV.

## Methods

### Design and study sample

In 2001, 48 schools from Hedmark and Telemark counties (24 schools in each county) were randomly selected and invited to participate in the FVMM research project (cohort I), and 19 schools from each county agreed to participate. All 6^th ^and 7^th ^graders (age 10-12) in these 38 schools were invited to take part in a questionnaire survey (which was the baseline survey for the FVMM intervention project) [16-18]. These 38 schools were re-contacted in 2008 and invited to once more participate in a similar survey among 6^th ^and 7^th ^graders (cohort II). At that time 27 schools agreed to participate, and all 6^th ^and 7^th ^graders in these 27 schools were invited to take part in the survey. The study sample of these two repeated cross-sectional studies includes 6^th ^and 7^th ^graders from both 2001 and 2008 at these 27 schools. Both studies were conducted in the September month. During this period there has been some changes in FV availability at some of the schools. A subscription program was implemented nation-wide in 2003, and all elementary schools are eligible to participate. This subscription program offers subscribing pupils one fruit or vegetable per day at schools taking part in the program. The cost of the subscription, covered by the parents, was 2.50 NOK per day (approximately €0.30). From autumn 2007, an official free school fruit program (without parental payment) was implemented in all secondary elementary schools (grades 8-10) and all combined schools (grades 1-10). Therefore, in 2001 none of the schools included in this study had any organized FV program, but in 2008, only 5 schools had a free FV program, 10 schools had a FV subscription program and 12 schools had no FV program. These nation-wide school fruit scheme has recently been evaluated within the FVMM project [19]. Research clearance was obtained from The Norwegian Social Science Data Services.

### Instrument

A questionnaire was completed by the pupils in the classroom in the presence of a trained project worker. One school-lesson (45 minutes) was used to complete the questionnaire. The FV intake among the pupils was assessed by the following four frequency questions; how often do you eat 1) vegetables for dinner, 2) other vegetables (e.g., carrot for school lunch), 3) apples, oranges, pears or bananas, 4) other fruits or berries. The response categories for all four questions had 10 alternatives ranging from 'never' = 0 to 'several times a day' = 10, giving a scale ranging from 0 to 40 times per week. In a sample of 114 6^th ^grade pupils, the test-retest correlation of this scale was 0.75 [20]. The correlation between the scale and a validation method (7 day food diary) was 0.32 in a separate validation study of 85 6^th ^grade pupils, a correlation which is similar to what have been found in other studies among the same age group [20].

The potential determinants, accessibility and preferences, were assessed by respectively five and four statements in the questionnaire with response alternatives ranging from 'I fully disagree' (value = -2) to 'I fully agree' (value = 2). The scores of these questions were summed. Preference had a possible range from -8 to 8 and was assessed by the following statements: 'Fruits and vegetables make my meals taste better', 'I really like raw vegetables', 'Fruits are among the best (foods) I know' and 'Fruits and vegetables are very suitable as snacks'. Perceived accessibility at home had a possible range from -10 to 10 and was assessed by: 'At home we usually have fruits and vegetables in the refrigirator', 'At home I am allowed to eat fruits and vegetables whenever I want', 'Mother or father do sometimes cut fruits and vegetables for me as a snack', "At home we usually have vegetables for dinner every day" and 'At home we usually have fruits available in a (fruit-) bowl'. These scales have been analysed for reliability, with test-retest correlations of 0.66 (accessibility) and 0.74 (preference) [21].

The pupils reported their own gender. After completing the questionnaire the pupils received a parent questionnaire to bring home to their parents for one of the parents to complete. The parents educational level was assessed individually by the parent answering the question: "What level of education do you have?". The question had four response alternatives: 'elementary school', 'high school', 'college or university (3 years or less)', and 'college or university (more than 3 years)'. This variable was dichotomized (lower: having no college or university education/higher: having attended college or university). The majority of those who completed the parental questionnaire were mothers (81.9%).

### Statistical analyses

**Figure 1:**
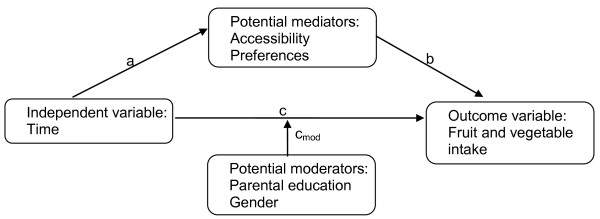
**Model of mediation and moderation of changes in FV intake over time**.

**Figure 2:**
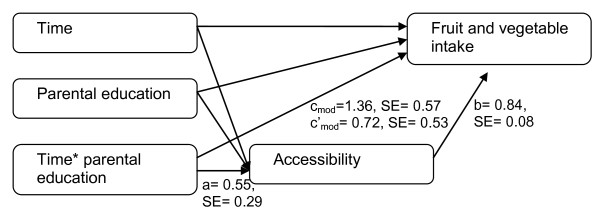
**Model of mediation of accessibility on moderated effect of SES on changes in FV intake**.

**Figure 3:**
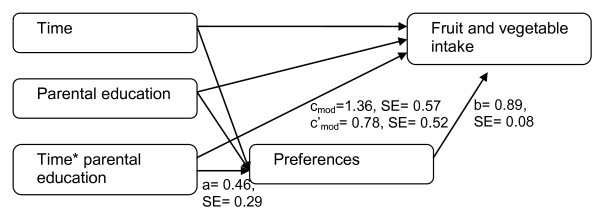
**Model of mediation of preferences on moderated effect of SES on changes in FV intake**.

Descriptive analyses were conducted by using one-way ANOVA in SPSS 14 (Table 1 and 2). The main analyses conducted were multilevel linear regression analyses using MLWin (version 2.02). We defined two levels in our multilevel analyses (1) student and (2) school. All models included time (2001 vs. 2008), gender, parental education and also whether the school participated in any school fruit program, as independent variables or covariates. First we calculated the total effect of time on FV intake (c-coefficient) (Figure 1). Second, the mediated effect of accessibility and preferences of the changes in FV intake over time were examined by using the products of coefficient method [22]. In this method, first the effect of time on the theoretical mediators accessibility and preferences is calculated (a-coefficient), followed by the calculation of the association of the theoretical mediators (i.e. accessibility and preferences) on FV intake after controlling for time (b-coefficient). The mediated effect is the product of the a- and b coefficient (a*b) and provides an estimate of the relative strength of the mediation effect. The Sobel test was used to assess the statistical significance of a mediating effect by dividing the products-of coefficients (a*b) by its standard error SE_ab _= √((a^2^*SE_b_^2^)+(b^2^*SE_a_^2^)). Third, in order to examine whether the trend in FV intake was different for different SES or gender groups (see Figure 1), we tested the moderated effect of parental education and gender on the changes in FV intake over time, by including two interaction terms ((1) time* parental_education and (2) time*gender)) into the first regression analyses (c_mod_-coefficient). A significant interaction term would indicate different changes in FV intake over time for the different subgroups. Fourth, in order to investigate the underlying reason for a possible interaction with SES or gender, a test of mediation of a moderating effect was conducted where it is assumed that the interaction predicts a mediator which predicts the outcome [23]. In other words, we examined whether a possible SES or gender disparity in changes in FV intake over time could be explained by a SES or gender disparity in changes in the potential mediators over time (see Figure 2 and 3). First we calculated the effect of the interaction terms (i.e. time*education_parents and time*gender) on the theoretical mediators (i.e. accessibility and preferences) (a_mod_-coeffient). Second, we calculated the effect of the theoretical mediators on FV intake when adjusted for the interaction terms between the moderator and independent variable (i.e. time*education_parents and time*gender) and the interaction terms between the moderator and the mediator variable (i.e. accesibility*parental_education and accessibility*gender or preference*parental_education and preference*gender) (b_mod_-coefficient). The mediation of a moderation effect can be estimated by the product-of-coefficient test (a_mod_*b_mod_) and its significance can be estimated by dividing it by its standard error (SEa_mod_b_mod _= √((a_mod_^2^*SEb_mod_^2^)+(b_mod_^2^*SEa_mod_^2^)). An examination of the residuals did not reveal unacceptable departures from normality. Since interaction terms have less power, p values, as an indicator of the significance, of interaction terms are recommended to be set at 0.10 [24]. All analyses in the current paper have been adjusted for whether the pupils were in schools participating in the fruit program or not. Attrition analysis were conducted, comparing the pupils at the 27 schools included in the study sample with the pupils at the 11 schools participating in 2001 but not in 2008, regarding gender, parental education, FV intake, accessibility and preferences. For the analysis t-tests were used for continous variables and χ^2 ^statistics were used (categorical data). No significant differences between the study sample and pupils at schools that did not participate in 2008 were found.

**Table 1:** Characteristics of the study population, FV intake and determinants of FV intake in the 2001 and 2008 survey

	2001		2008	
	
	n	Mean (95% CI)	n	Mean (95% CI)
Schools	27		27	
Pupils	1488		1339	
Gender				
Boys	748 (50.3%)		630 (47.9%)	
Girls	738 (49.7%)		684 (52.1%)	
Grade				
6th	782 (52.6%)		686 (51.3%)	
7th	706 (47.4%)		652 (48.7%)	
Parents	1230		996	
Parental educational level				
EDU high	511 (42.2%)		527 (53.6%)	
EDU low	699 (57.8%)		457 (46.4%)	
Intake and determinants				
FV intake	1442	14.2 (13.8 - 14.6)	1263	13.9 (13.5 - 14.2)
Accessibility	1487	4.0 (3.8 - 4.2)	1333	5.1 (4.9 - 5.3)
Preferences	1480	2.7 (2.5 - 2.9)	1320	3.1 (2.9 - 3.2)

CI, confidence interval. FV intake, fruit and vegetable intake. EDU high, higher parental education. EDU low, lower parental education

**Table 2:** Comparisons of the 2001 and 2008 cohorts on the four separate FFQ items

Question	n	Mean	Standard Deviation	p-value*
How often do you eat vegetables for dinner?				
2001	1476	3.8	2.2	
2008	1321	3.9	2.1	0.439
How often do you eat other vegetables?				
2001	1462	2.8	2.5	
2008	1292	2.5	2.3	0.003
How often do you eat apples, oranges, pears or bananas?				
2001	1470	4.6	2.7	
2008	1317	4.9	2.5	0.009
How often do you eat other fruits and berries?				
2001	1466	3.0	2.5	
2008	1294	2.7	2.2	< 0.001

* One-Way ANOVA

## Results

A total of 1488 pupils (out of 1727 eligible; 86%) in 2001 and 1339 pupils (out of 1712 eligible; 78%) in 2008 completed the questionnaire and brought home a parent questionnaire to be completed by one of their parents. For respectively 1230 and 996 pupils, one of their parents completed the parent questionnaire. Descriptions of the samples in 2001 and 2008 are presented in Table 1.

### Changes in FV intake, accessibility and preferences over time

Table 1 shows the changes in FV intake, accesibility and preferences of FV over time. A decrease in FV intake from 14.2 to 13.9 times/week among 6^th ^and 7^th ^graders at the 27 schools was observed from 2001 to 2008 (c = -0.55, SE = 0.29, p = 0.06). At the same time mean scores in both accessibility and preferences significantly increased from 4.0 to 5.1 (a = 1.08, SE = 0.15, p < 0.001) and from 2.7 to 3.1 (a = 0.31, SE = 0.15, p < 0.05), respectively. Analyzing the four items in the FV scale separately, the consumption of 'vegetables for dinner' (3.8 vs. 3.9 times/week, p = 0.44) and 'apples, oranges, pears and bananas' (4.6 vs. 4.9, p = 0.009) increased, while the intake of 'other vegetables' (2.8 vs. 2.5, p = 0.003) and 'other fruits and berries' (3.0 vs. 2.7, p < 0.001) decreased from 2001 to 2008 (Table 2).

### Mediated effect of accessibility and preferences on changes in FV intake over time

Mediation analyses showed that both changes in accessibility (ab = 0.89, SE = 0.13, p < 0.001) and changes in preferences (ab = 0.25, SE = 0.12, p < 0.05) suppressed the changes in FV intake over time (Table 3). Suppressor effects, also called inconsistent mediated effects, are mediated effects with a different sign than the direct effect in a model. This inconsistent mediator suppresses the total effect. In other words, the decrease in FV intake over time would have been higher if the accessibility and preference of FV had not increased.

**Table 3:** Trend in FV intake and the mediated effect of preferences and accessibility on this trend

	c (SE)	c' (SE)	a(SE)	b (SE)	ab (SE)
FV intake	-0.55 (0.29)‡				
Accessibility		1.42 (0.26)***	1.08 (0.15)***	0.82 (0.04)***	0.89 (0.13)***
Preferences		0.83 (0.26)**	0.31 (0.15)*	0.83 (0.04)***	0.25 (0.12)*

c = total change in FV intake over time; c' = change in FV intake over time when adjusted for changes in the mediator; a = change in mediator over time; b = association between mediator and FV intake when adjusted for changes over time; ab = mediated effect using the product of coefficient test. SE, standard error. All analyses are adjusted for clustering effects and time, gender, parental education and treatment condition. ‡ p < 0.10; * p < 0.05; **p < 0.01; ***p < 0.001

### Moderated effect of parental education and gender on changes in FV intake over time

During this time period the proportion of parents with higher education increased from 42.2% to 53.6% (Table 1). The multilevel linear regression analysis on FV intake showed a significant interaction between parental education level and time (c_mod _= 1.36, SE = 0.57, p = 0.01) (Figure 2 and 3). Subgroup analyses showed that FV intake decreased among pupils of parents with a low education from 13.9 times/week in 2001 to 12.6 times/week in 2008 (c = -2.72, SE = 0.73, p < 0.001), and slightly increased among pupils of parents with higher education from 14.8 times/week in 2001 to 15.0 times/week in 2008 (c = 1.08, SE = 0.77, p = 0.16). These results indicate that SES disparity of FV intake increased over time in which lower SES children had a higher decrease in FV intake over time compare to a more stable intake among higher SES children. No significant interaction between time and gender was found (c_mod _= 0.31, SE = 0.57, p = 0.59).

### Mediation of a moderated effect of parental education and gender on changes in FV intake

Parental education moderated the changes in accessibility (a_mod _= 0.55, SE = 0.29, p = 0.06) (Figure 2) and preferences (a_mod _= 0.46, SE = 0.29, p = 0.11) (Figure 3) over time (Table 4). Subgroup analyses showed that children with high educated parents had a steeper increase in accessibility (a_mod _= 1.17, SE = 0.38, p < 0.00) and preferences (a_mod _= 0.14, SE = 0.38, p = 0.70) than children with low educated parents (a_mod _= 0.82, SE = 0.40, p < 0.05; a_mod _= 0.05, SE = 0.39, p = 0.90). These results indicate that SES disparity in FV accessibility and preferences increased over time in which higher SES children had a higher increase in accessibility and preferences than lower SES children. Both accessibility (b_mod _= 0.84, SE = 0.08, p < 0.001) and preferences (b_mod _= 0.89, SE = 0.08, p < 0.001) were significantly independent associated with FV intake when adjusted for the independent variables and interaction term between time and parental education. A mediating effect of accessibility (ab_mod _= 0.46, SE_mod _= 0.24, p = 0.05) and preferences (ab_mod _= 0.41, SE_mod _= 0.26, p = 0.11) on the moderating effect of SES on FV intake was found (Table 4). This indicates that the increasing SES disparity in changes in FV intake over time could partly be explained by an increasing SES disparity in accessibility and preferences of FV over time. No significant mediation of accessibility (ab_mod _= 0.05, SE = 0.25, p = 0.84) and preferences (ab_mod _= 0.22, SE = 0.26, p = 0.39) was found on the moderating effect of gender on changes in FV intake over time.

**Table 4:** Mediated effect of accessibility and preferences on moderated effect of SES on FV intake trend

	c_mod _(SE)	c'_mod_(SE)	a_mod _(SE)	b_mod _(SE)	ab_mod _(SE)
FV intake	1.36 (0.57)				
Accessibility		0.72 (0.53)	0.55 (0.29) ‡	0.84 (0.08)***	0.46 (0.24)‡
Preferences		0.78 (0.52)	0.46 (0.29)	0.89 (0.08)***	0.41 (0.26)

the interaction term (time*parental education) is the independent variable.

c_mod _= effect of parental education on the changes in FV intake over time; c'_mod _= effect of parental education on the changes in FV intake over time adjusted for changes in the mediator; a_mod _= effect of parental education on the changes in the mediators over time; b_mod _= association between mediators and FV intake when adjusted for the moderator; ab_mod _= mediated effect of mediators on the effect of parental education on the change in FV intake over time. SE, standard error. All analyses are adjusted for clustering effects and time, gender, parental education and treatment condition. ‡ p < 0.10; *p < 0.05; **p < 0.01; ***p < 0.001

## Discussion

The present study shows an increase in SES disparity in FV intake among 10-12 year olds from 2001 to 2008, wherein low SES children had a steeper decline in FV intake than high SES children. This increase in SES disparity was partly mediated by an increasing SES disparity in accessibility and preferences, wherein high SES children had a steeper increase in accessibility and preferences than low SES children. Moreover, increases in accessibility and preferences over time were found to suppress the decrease in FV intake over time. This indicates that the decrease in FV intake would have been higher if accessibility and preferences had not increased over time. This points out that accessibility and preferences are relevant determinants of FV intake which confirms the findings of previous research [11]. The gender disparity regarding FV intake did not change from 2001 to 2008.

Studies on how the socioeconomic disparities in eating behaviors have developed over time are limited. Previous observational studies on dietary behavior reveal that healthy eating habits decreases as the adolescents get older, and that the SES disparities increased [2]. In a recent review, adolescents' of lower SES had poorer diets compared to adolescents of higher SES in 14 out of 16 studies [25]. The authors concluded that the observed associations between SES and eating habits among adolescents seemed less robust than the association between SES and eating habits among adults. Within the FVMM study previous longitudinal analysis has shown an increased socioeconomic disparity in FV intake among adolescents as they aged from 12.5 (year 2002) to 15.5 years (year 2005). The difference in the socioeconomic disparities in FV intake among these adolescents increased from 1.3 times/week in 2002 (age 12.5, p = 0.03) to 2.4 times/week in 2005 (age 15.5, p < 0.001) [8]. The present study shows that the SES disparities in FV intake within the same age group (10-12 year olds) increased from 0.9 in 2001 to 2.4 times/week in 2008 (data not shown). A study on similar trends in SES differences in FV intake among Dutch schoolchildren recently reported that girls of mothers with lower educational level reported lower fruit intake in 2009 compared to 2003 (unpublished work by Fischer C, Brug J, Tak N and Te Velde S). This shows that there probably is a trend in the society towards greater SES disparities with regard to FV intake in two highly developed countries, Norway and the Netherlands. This trend might also explain at least parts of the age-trend reported above. Whether it is the increasing age or development in time which contributes most to these increased socioeconomic disparities needs further investigation.

Bere and colleagues [8] have previously reported that SES disparities regarding perceived accessibility and preferences for FV explains most of the SES differences observed in FV intake. The present study adds to this by showing that changes in accessibility and preferences also mediate parts of the increasing SES disparities regarding changes in FV intake within the same age group from 2001 to 2008, wherein high SES children had a higher increase in accessibility and preferences than low SES children.

This increasing disparities regarding SES differences in adolescents FV intake, accessibility and preferences of FV is the opposite trend of what the Norwegian government has been aiming for [14]. One effort of the Norwegian government in trying to reduce social disparities in health is a free school fruit scheme implemented at all secondary schools (grades 8-10) and all combined schools (1-10) from fall 2007. It is now legally established that all pupils in secondary schools receive a free fruit at school every school day [26]. This nation-wide free school fruit scheme has recently been evaluated within the FVMM project [19], using the same data set as the present study. A greater increase in fruit intake within the schools participating in the program (i.e. schools with grades 1-10, 5 out of the 27 schools) compared to the other schools was observed. In addition, it was indicated that the free fruit scheme was effective in increasing fruit intake in all groups at these schools (including boys and pupils of lower SES) as the interactions between intervention effect and gender and SES were not significant. However, this effect was probably not sufficient in order to limit the increasing socioeconomic disparities in the pupils' frequency of FV consumption, as presented in the present paper, during the same period. This might be because only a limited number of schools (5 out of 27) received free fruit. The results of Bere et al. [19] also showed that in schools not participating in any FV program the percentage of pupils eating FV at school 4-5 days per week increased by 12 percentage points among the pupils of high SES whereas there were no changes in the low SES group from 2001 to 2008, adding support to our findings in the present study.

The contradicting results on the time trend in FV intake found in the current study, compared to Bere et al. [19] may be explained by the methods used to assess FV intake. In the study referred to above, a 24-h recall was used to assess FV intake in order to assess the effect of the school fruit programs, while the current study used FFQ's to assess FV intake to be able to relate FV intake to accessibility and preferences. In the current study however, we observed an increase in intake of apples, oranges, pears and bananas from 2001 to 2008 (Table 2). This may reflect an increased intake of fruits at school, as the school fruit scheme mostly serves these kinds of fruits. The decline reported in consumption of other vegetables and other fruits and berries assessed by the FFQ's (Table 2) might be due to these questions being somehow vague. However, during the last decade there has been considerable publicity by the Norwegian government on promoting FV intake, also among children and adolescents. This might have contributed that the pupils of 2008 were more aware and able to report their FV intake compared to the 2001 pupils. A hypothesis might be that pupils of 2008 report to eat more FV on those occasions where they know they are served FV (school and dinner) and being less likely to over report on vague items such as 'other vegetables/fruits'.

A strength of the present study is that it includes pupils at two time points (2001 and 2008) from the same 27 randomly selected schools. There are some limitations to this study. First, the schools included were from only 2 out of 19 Norwegian counties. However, since the attrition analysis showed no significant differences between study sample and the 11 schools not participating in 2008 and since Norway is a rather homogenous country the results from this study can probably be generalized to all Norwegian counties. Second, the 2008 sample had a higher proportion of high SES pupils compared to the 2001 sample (i.e. in 2008 more of the parents reported a higher education level). A higher proportion of high SES pupils in the study sample for 2008 probably reflect the increasing educational level in the population [27]. However, it may also be that the proportionally less pupils in 2008 compared to 2001 had parental reported SES data and that research suggests that parents of high SES groups are more likely to respond to research requests. Third, most of the parents who filled out the questionnaires, and who's educational level was used to assess SES, were mothers (81.9%). This might not reflect the all over family SES completely.

Our findings indicate the need for further research and enhanced efforts to reduce the socioeconomic disparities in adolescent FV intake. A next question would be to ask: How can we improve the accessibility at home and preferences among children and adolescents of low SES groups? There is clearly a need for intervention studies on increasing the children and adolescents accessibility and preferences, especially among those of the lower SES groups.

## Conclusions

The results show an increase in social disparities from 2001 to 2008 in FV intake, accessibility and preferences of FV among adolescents aged 10-12 years. Accessibility and preferences mediated parts of the increase in SES disparity in FV intake.

## Abbreviations

FV: fruits and vegetables; GDP: gross domestic product; SES: socioeconomic status; FVMM: Fruits and Vegetables Make the Marks; ANOVA: Analysis of variance between groups; SPSS: Statistical Package for the Social Sciences; SE: standard error; CI: confidence interval.

## Competing interests

The authors declare that they have no competing interests.

## Authors' contributions

KIK conceived the study in 2001. EB conceived the study in 2008. EB and MH designed the current study. MH and MMS analyzed the data and all authors contributed to interpretation. MH drafted the manuscript and MMS, EB and KIK critically revised it. All authors approved of the final manuscript.

## Funding

The project was funded by the Norwegian Research Council (Grant number 185817/V50). The contribution of MMS was funded by Netherlands Organization for Health Research and Development (Grant number 121520002).
